# Supplementary figures and images for: Phylogenomic Analyses of the Tenthredinoidea Support the Familial Rank of Athaliidae (Insecta, Tenthredinoidea)
Source: Insects. 2022 Sep 21;13(10):858. doi: 10.3390/insects13100858 (PMC9604231; doi:10.3390/insects13100858)

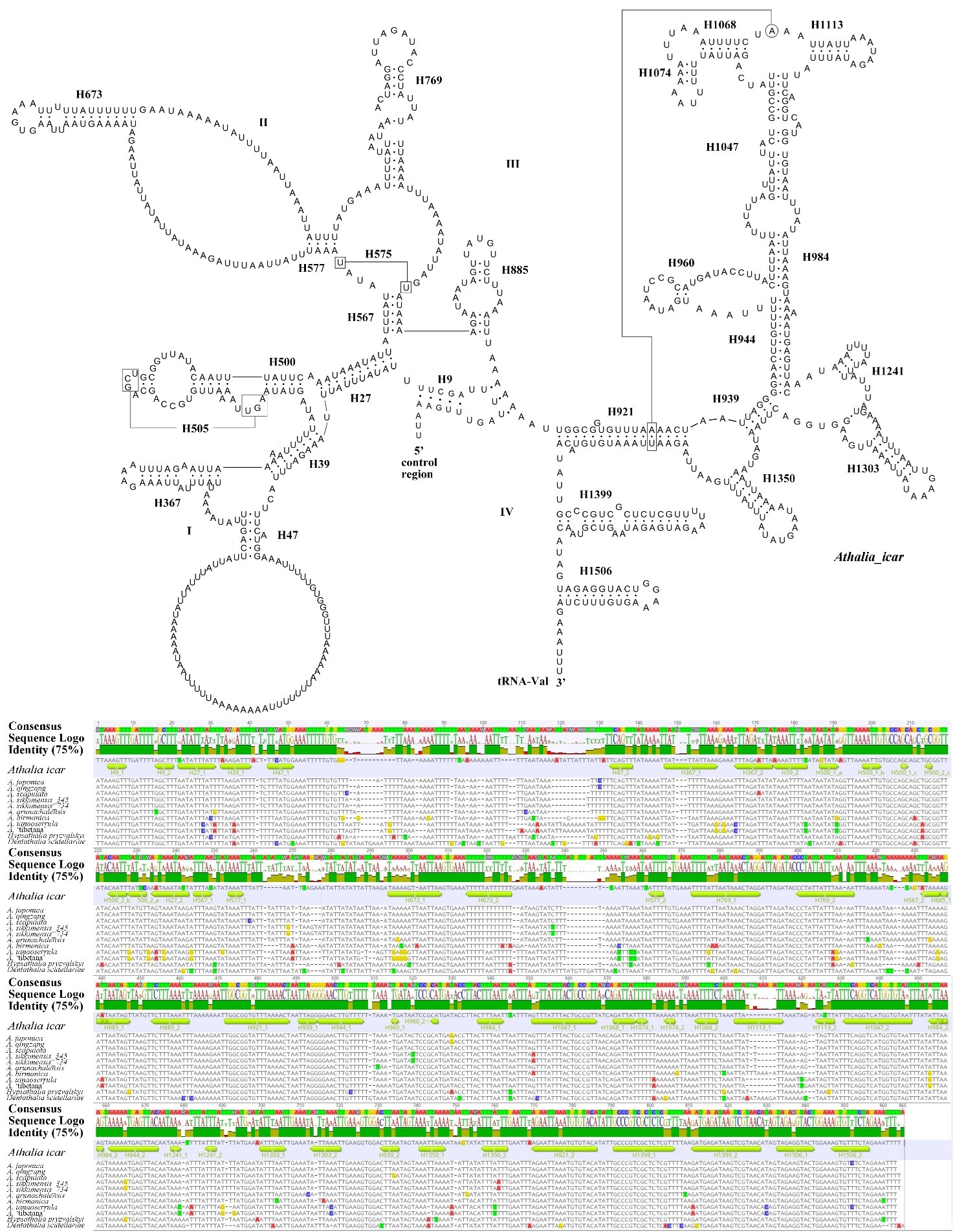

Supplement: Supplementary file 1 [file insects-13-00858-s001.zip › Figure S1.jpg]

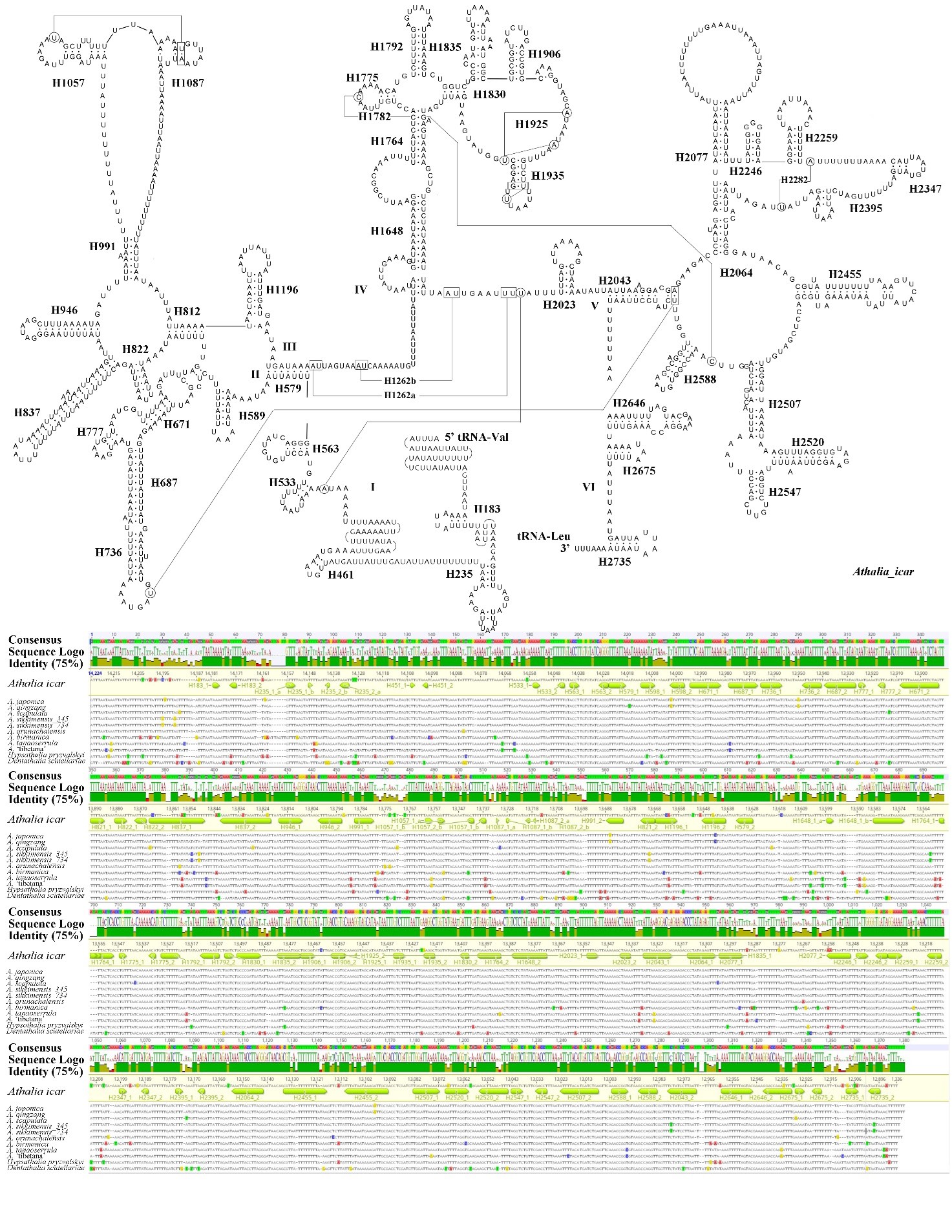

Supplement: Supplementary file 1 [file insects-13-00858-s001.zip › Figure S2.jpg]
